# Diet analysis by next-generation sequencing indicates the frequent consumption of introduced plants by the critically endangered red-headed wood pigeon (*Columba janthina nitens*) in oceanic island habitats

**DOI:** 10.1002/ece3.773

**Published:** 2013-09-19

**Authors:** Haruko Ando, Suzuki Setsuko, Kazuo Horikoshi, Hajime Suzuki, Shoko Umehara, Miho Inoue-Murayama, Yuji Isagi

**Affiliations:** 1 Laboratory of Forest Biology, Division of Forest and Biomaterials Science, Graduate School of Agriculture, Kyoto UniversityKyoto, 606-8502, Japan; 2 Department of Forest Genetics, Forestry and Forest Products Research InstituteTsukuba, Ibaraki, 305-8687, Japan; 3 Institute of BoninologyChichijima, Ogasawara, Tokyo, 100-2101, Japan; 4 Wildlife Research Center, Kyoto UniversityKyoto, 606-8203, Japan

**Keywords:** Conservation, diet analysis, DNA barcoding, next-generation sequencer, oceanic islands, red-headed wood pigeon

## Abstract

Oceanic island ecosystems are vulnerable to the introduction of alien species, and they provide a habitat for many endangered species. Knowing the diet of an endangered animal is important for appropriate nature restoration efforts on oceanic islands because introduced species may be a major component of the diets of some endangered species. DNA barcoding techniques together with next-generation sequencing may provide more detailed information on animal diets than other traditional methods. We performed a diet analysis using 48 fecal samples from the critically endangered red-headed wood pigeon that is endemic to the Ogasawara Islands based on chloroplast *trn*L P6 loop sequences. The frequency of each detected plant taxa was compared with a microhistological analysis of the same sample set. The DNA barcoding approach detected a much larger number of plants than the microhistological analysis. Plants that were difficult to identify by microhistological analysis after being digested in the pigeon stomachs were frequently identified only by DNA barcoding. The results of the barcoding analysis indicated the frequent consumption of introduced species, in addition to several native species, by the red-headed wood pigeon. The rapid eradication of specific introduced species may reduce the food resources available to this endangered bird; thus, balancing eradication efforts with the restoration of native food plants should be considered. Although some technical problems still exist, the *trn*L approach to next-generation sequencing may contribute to a better understanding of oceanic island ecosystems and their conservation.

## Introduction

Oceanic island ecosystems are a high biodiversity conservation priority because of their high endemism of fauna and flora owing to long periods of evolution under highly isolated conditions (Whittaker 2007), but they are vulnerable to human disturbances, such as deforestation and the introduction of invasive species (Kachi 2010; Kawakami 2010). For example, with regard to bird species, 431 endangered species inhabit oceanic islands, such as honeycreepers in Hawaii and flightless birds in New Zealand (BirdLife International 2008). The diet of an endangered species is an important issue to consider when restoring native forests and eradicating introduced species; some introduced species have become essential components of the current ecosystem, which complicates this issue (Kawakami 2008). For example, 90% of the diet of the endangered Ogasawara buzzard *Buteo buteo oyoshim* is thought to be composed of introduced animals (e.g., the black rat *Rattus rattus* and the green anole *Anolis carolinensis*); therefore, the rapid eradication of these introduced species may negatively affect the buzzard population (Kato and Suzuki 2005). Thus, a detailed understanding of the food web, including native and introduced species, is required for the conservation of oceanic island ecosystems.

**Figure 1:**
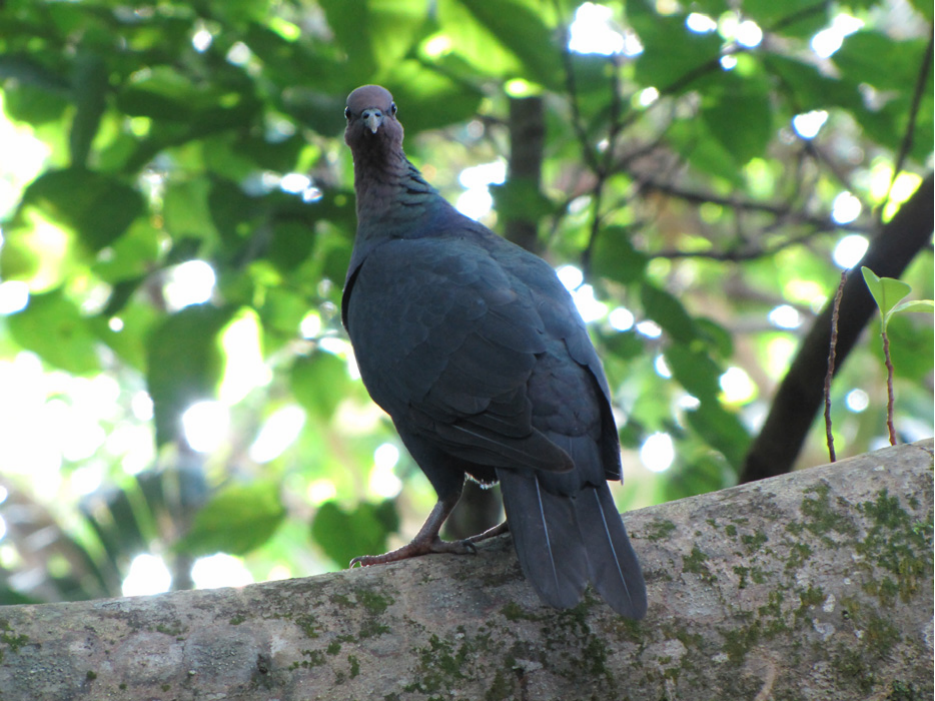
Red-headed wood pigeon *Columba janthina nitens*.

The red-headed wood pigeon *Columba janthina nitens* (Fig. 1) is a critically endangered subspecies endemic to the Ogasawara Islands, a chain of oceanic islands located 1000 km south of the main islands of Japan (Fig. 3). The present population of this pigeon is thought to include approximately 100 individuals, according to observation records (Horikoshi 2008), and this species is listed as critically endangered on the Japanese Red List (Environmental Agency of Japan 2006). The red-headed wood pigeon is thought to be a seedeater (Takano et al. 1995) based on direct observations (feeding on *Elaeocarpus photiniifolius*, *Neolitsea boninensis*, *Melia azedarach* and *Ficus microcarpa,* etc. were recorded), similar to other *Columba* species (Gibbs et al. 2001). To maintain the foraging habitat of the pigeon, a forest must maintain its species diversity and supply seeds throughout the seasons (Kawakami 2008). However, the native forest of the Ogasawara Islands has been destroyed because of human settlements in the 19th century and World WarII (Kachi 2010; Kawakami 2010). Furthermore, several introduced plants, such as *Bischofia javanica*, *Ficus microcarpa*, *Leucaena leucocephala* and *Morus australis*, have expanded their populations and generate a large abundance of fruit (Ecological Society of Japan 2002; Toyoda 2003; Hata et al. 2010; Tanaka et al. 2010). The red-headed wood pigeon may frequently consume introduced species to fulfill a lack of native food resources, and these species will be eradicated during nature restoration projects in the near future.

Fecal analysis via a DNA barcoding technique depends on a taxon identification system using a standardized DNA region (Hebert and Gregory 2005). This method may be an effective noninvasive approach for studying the diet of the red-headed wood pigeon. Thus far, dietary analyses of this animal have been carried out mainly by direct observation (Kanto Regional Forestry Office 2005, 2006; Kanto Regional Environmental Office 2011) and microhistological analysis (Shibazaki and Hoshi 2006). However, the existing information on the diet of this species is fragmented because of the difficulty of continuous observation and species identification of digested food items in pigeon stomachs. Via the DNA barcoding method, fecal sequencing using a next-generation sequencer (NGS) can provide large amounts of sequence data without a cloning step. Thus, the time and cost of analysis are reduced, and more detailed animal diet information can be collected (Valentini et al. 2009a,b; Pompanon et al. 2012). Although food DNA in fecal samples is often degraded, the universal short barcode primer *trn*L g-h (Taberlet et al. 2007) can effectively amplify plant DNA, as shown in previous herbivore diet analyses (Valentini et al. 2009a; Kowalczyk et al. 2011; Raye′ et al. 2011).

The higher resolution resulting from the DNA barcoding approach via NGS in comparison with traditional microhistological analysis has been noted in several previous studies (Pompanon et al. 2012). However, there have been few attempts to compare the two methods using the same sample set. Soininen et al. (2009) suggested a comparison of the high-resolution DNA barcoding approach with a microhistological analysis for the dietary analysis of stomach contents. The resolution difference between the two methods is unknown for fecal samples, in which food items are more degraded than stomach contents.

The aims of the present study were to estimate the usefulness of the DNA barcoding approach combined with NGS for the red-headed wood pigeon fecal analysis, to compare the resulting resolution to that of the microhistological analysis using identical sample sets, and to determine the current dietary composition (regarding whether this diet includes introduced species) of this pigeon.

## Materials and Methods

**Figure 2:**
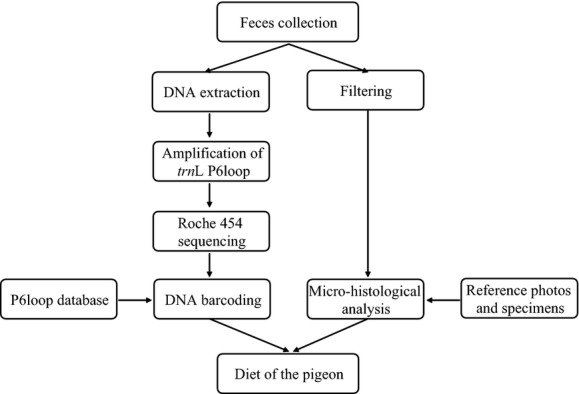
General outline of the diet analysis for the red-headed wood pigeon.

Figure 2 provides a general outline for the dietary analysis of the red-headed wood pigeons analyzed in this study. We conducted both NGS DNA barcoding and microhistological analysis using the same sample set and compared the results of the two methods. After determining the diet of the pigeons, we performed additional analyses using the NGS DNA barcoding data.

### Development of the *trn*L reference database

**Figure 3:**
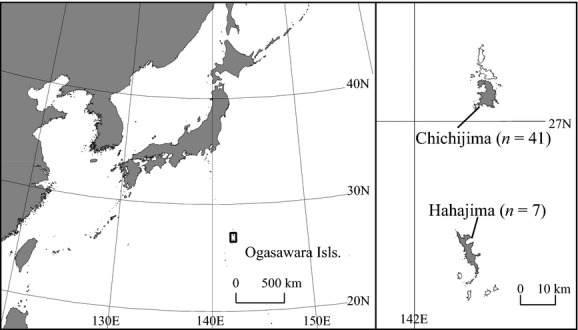
Sampling locations of the study. The left map shows the location of the Ogasawara Islands. In the right map, the gray-colored islands are the sampling sites for Chichijima and Hahajima. The numbers in parentheses are the fecal sample sizes.

Samples from 222 seed plant species were collected from Chichijima and Hahajima in the Ogasawara Islands (Fig. 3). We covered 93% of the woody seed plants (118 species) described in Toyoda (2003). Other woody and herbaceous plants were collected by searching around the pigeon habitat. The samples were stored at −30°C before the DNA extraction, which was performed using a DNeasy Plant Mini Kit (Qiagen, Venlo, Netherlands), following the manufacturer's instructions. The universal primer pair c–d (Taberlet et al. 1991) was used for PCR amplification of whole chloroplast *trn*L (UAA) introns (c: 5′-CGAAATCGGT AGACGCTACG-3′; d: 5′-GGGGATAGAGGGACTTGAA C-3′). Each 10 μL of the total reaction mixture volume contained 5 ng of extracted DNA, 5 μL of 2 × Multiplex PCR Master Mix, and 0.2 μmol/L of each primer pair. The PCR conditions were as follows: denaturation for 15 min at 95°C; 35 cycles of 30 s at 94°C, 1.5 min at 57°C, and 1 min at 72°C; and a final 10 min extension at 72°C. Cycle sequencing was performed with a Big Dye Terminator v1.1 Cycle Sequencing Kit (Applied Biosystems, Foster City, CA) according to the standard protocol. The cycle sequencing products were visualized by the ABI PRISM 3100 Genetic Analyzer (Applied Biosystems). To build the reference database, P6 loop sequences (Taberlet et al. 2007) were extracted from the entire *trn*L (UAA) intron sequence. The *Passiflora edulis* sequence was retrieved from GenBank and added to the database.

### Fecal sampling and DNA extraction

We collected 48 fecal samples from the red-headed wood pigeons in Chichijima and Hahajima from September 2009 to May 2011 (Fig. 3). Sampling on Chichijima was carried out continuously from September 2010 to May 2011. Of these samples, 35 were collected after directly observing pigeon elimination. The remaining 13 were collected from areas around nests and roosts, considering size, shape, and contents (mainly crushed seeds). The collected feces were stored at −30°C before DNA extraction. DNA was extracted from 20 mg of fecal dry weight using a DNeasy Plant Mini Kit (Qiagen). The remainder of each sample (more than half) was used for microhistological analysis.

### PCR amplification and sequencing from fecal DNA

To confirm that the 13 samples collected around the nests and roosts belonged to the red-headed wood pigeon, we amplified a portion of the mitochondrial COI region sequences (290 bp) with the primer pair *PSF* (5′-AAC CCGGCACCCTTCTAGGAGACGA-3′) and *PSR* (5′-ACCAGCTAGAGGTGGATAAACAGTT-3′). The primers were designed to avoid amplifying the mitochondrial DNA of other native bird species in the Ogasawara Islands (e.g., the brown-eared bulbul *Hypsipetes amaurotis* and scaly thrush *Zoothera dauma*; The Ornithological Society of Japan 2012) besides the red-headed wood pigeon. Each 10 μL of total reaction mixture volume contained 5 ng of extracted DNA, 0.05 μL of ExTaq (Takara), 1 μL of 10 × ExTaq Buffer, 0.8 μL of dNTPs, and 0.12 μmol/L per primer pair. The PCR conditions were as follows: denaturation for 2 min at 94°C; 40 cycles of 15 s at 94°C, 30 s at 52°C, and 1 min at 72°C; and a final cycle of 5 min at 72°C. We checked for the presence of a PCR product of suitable length by electrophoresis on a 1.5% agarose gel.

The universal primer pair *g* (5′-GGGCAATCCTGAGCCAA-3′) and *h* (5′-CCATTGAGTCTCTGCACCTATC-3′; Taberlet et al. 2007) was used to amplify the *trn*L P6 loop. The forward primer was tagged with a multiplex identifier (MID, Roche Diagnostic) to identify the resulting sequences from each sample. PCR amplification was conducted using a Qiagen Multiplex PCR kit (Qiagen). Each 25 μL of total reaction mixture volume contained 20 ng of extracted DNA, 15 μL of 2 × Multiplex PCR Master Mix, and 0.2 μmol/L of each primer pair. The PCR conditions were as follows: denaturation for 15 min at 95°C; 45 cycles of 30 s at 94°C, 1.5 min at 57°C, and 1 min at 72°C; and a final cycle of 10 min at 72°C. The PCR products were purified using exo/SAP (*exonuclease I* and *shrimp alkaline phosphatase*, Takara, Shiga, Japan and Promega, Madison, WI) and a High Pure PCR Products Purification Kit (Roche Diagnostic, Basel, Switzerland) and then quantified using a NanoDrop ND-1000 UV–Vis Spectrophotometer (NanoDrop Technologies, Wilmington, DE). The length of the PCR products was checked using a High Sensitivity DNA Kit (Agilent Technologies, Santa Clara, CA) to confirm that the targeted region sequences were correctly amplified and that short fragments were completely excluded during the purification process. Next, the PCR products were mixed such that approximately the same number of molecules from each fecal sample was included in each mix (Pegard et al. 2009). Large-scale pyrosequencing was carried out on a 454 GS Junior Sequencer (Roche Diagnostic) following the manufacturer's instructions.

### DNA barcoding

The resulting sequences were separated into each sample by MID tags. The sequences in each sample were assembled using the CAP Contig Assembly Program in BioEdit software (Hall 1999) for a 98% match. The short sequences (less than 40 bp) and contigs, which included fewer than four sequences in each sample, were excluded from DNA barcoding. The DNA barcoding for each contig was carried out using a local BLAST in BioEdit. We detected a plant within the P6 loop database that exhibited the highest score with low e-values (<1.0e–25) for each contig. If two or more taxa are assigned the same score for a given contig, the contig was assigned to the lowest taxonomic level that included both taxa.

### Microhistological analysis

We used the 47 fecal samples remaining after DNA extractions for microhistological analysis. All of one sample was used for DNA barcoding because of its small amount. We followed the modified method of Shibazaki and Hoshi (2006). The fecal samples were washed with water and filtered using a 0.5 mm screen. Any fragments remaining on the screen were examined under the microscope at 20 × magnification. Fragment identification was performed using reference photos, and seed specimens collected in the Ogasawara Islands.

### Data analysis

The discrimination rate for the P6 loop database at the three taxonomic levels (species, genus, and family) was calculated, as described by Raye′ et al. (2011). The discrimination rate for the species level (*R*s) was calculated by dividing the number of unique sequences by the number of species in the database. The discrimination rate at the genus level (*R*g) was calculated by dividing the number of genera with one or more unique sequences by the total number of genera. The rate of discrimination for the species in each family (*R*f) was calculated by dividing the number of unique sequences per family by the number of species in the family.

After DNA barcoding, we calculated the frequency of sequence reads (*F*_R_) and the frequency of the presence (*F*_P_) of each food item. To compare the resolution of DNA barcoding and microhistological analysis, the numbers of detected food plants per sample and frequency of each food item (number of samples in which the specific food item was observed divided by the total number of samples) were calculated. Each food plant was classified as native or introduced. Using the data from DNA barcoding, the following analyses were also performed. To estimate the monthly change in diet during the breeding season in Chichijima, the relative frequency of data for major food items during each month was calculated. To compare the diets of pigeons on Chichijima and Hahajima, we performed island-based (samples were pooled for each island) and sample-based analyses. In the island-based analysis, the frequency of reads and the relative frequency of presence data for each plant taxa were calculated. The diet composition differences were tested by Pearson's chi-square tests. In the sample-based analysis, nonmetric multidimensional scaling (NMDS) on the Chao similarity index (Chao et al. 2004) and an analysis of similarities (ANOSIM, Clarke 1993) on a Chao matrix (Doi and Okamura 2011) were performed by the vegan package in R (Oksanen et al. 2010) using the number of reads and the presence/absence data of each plant taxa. We also compared the diet composition and diversity (number of reads vs. presence/absence of each plant taxa) measurements in these analyses.

## Results

### Plant discrimination using the *trn*L P6 loop database

The *trn*L P6 loop was sequenced for 222 plant species belonging to 175 genera and 76 families (accession numbers: AB817341-AB817701). Of the 222 sequences of the P6 loop database, 167 unique sequences were found. The sequence fragment lengths ranged from 66 to 148 bp. The discrimination rate at the species level, or *R*s, was 75%, and the *R*g, or genus level, was 89%. All families were identified using the P6 loop. Discrimination rates within families ranged from 14% to 100% (Table 1 and see Appendix S1 for the correspondence between species and groups of species).

**Table 1:** Species discrimination rate (*R*f) for 38 families that have more than one species in the Ogasawara Islands database

Family	*N* species	*N* sequences	*R*f (%)
Agavaceae	3	2	67
Anacardiaceae	2	2	100
Apocynaceae	2	2	100
Aquifoliaceae	3	1	33
Asteraceae	9	8	89
Caprifoliaceae	2	2	100
Cyperaceae	12	8	67
Ebenaceae	2	1	33
Euphorbiaceae	5	5	100
Fabaceae	11	11	100
Lauraceae	7	1	14
Liliaceae	2	2	100
Malvaceae	3	2	50
Melastomataceae	2	1	50
Moraceae	9	2	20
Myrsinaceae	3	2	67
Myrtaceae	6	3	50
Nyctaginaceae	3	3	100
Oleaceae	3	3	100
Palmae	9	3	33
Pandanaceae	2	2	100
Piperaceae	2	2	100
Pittosporaceae	4	1	25
Poaceae	24	17	71
Ranunculaceae	2	2	100
Rosaceae	4	2	50
Rubiaceae	13	12	92
Rutaceae	9	4	44
Sapindaceae	2	2	100
Sapotaceae	3	1	33
Solanaceae	3	3	100
Stachyuraceae	2	1	50
Symplocaceae	2	1	50
Theaceae	2	2	100
Ulmaceae	2	2	100
Urticaceae	2	2	100
Verbenaceae	7	5	75
Zingiberaceae	3	3	100

### Resolution comparisons between DNA barcoding and microhistological analysis

All 13 samples collected around the nests and roosts were confirmed as originating from the red-headed wood pigeon by amplification using the primers *PSF* and *PSR*. The sequencing of 48 fecal samples yielded 35,666 reads, corresponding to an average of 743 ± 338 (SD) bp and ranging from 157 to 1747. From the results of the DNA barcoding with the P6 loop database, 44 plant taxa were detected from 32,291 reads (approximately 90% of the total sequences). Most of the reads that were not assigned to a specific plant taxa in the P6 loop database (3225 reads) were short or had a low frequency. Of the 32,391 reads, more than 70% belonged to *Morus australis* (36.58%) and Gr. Lauraceae1 (34.94%; Table 2), indicating their high consumption by pigeons and/or the high PCR amplification efficiency of these plants. The number of detected food plants per sample in the DNA barcoding (6.73 ± 2.70) was significantly greater than that obtained from the microhistological analysis (1.42 ± 0.62, *P* < 0.01, *t*-test). When comparing food items detected by DNA barcoding and microhistological analysis, Lauraceae (identified as *Neolitsea aurata* in the microhistological analysis), *Fagara boninsimae,* and *Planchonella* were frequently observed using both methods with similar frequencies of presence (Table 2). However, plants such as *Morus australis*, *Ficus,* and Poaceae were frequently observed using DNA barcoding only. Although they were identified only at low frequencies (observed in one sample each), *Distylium lepidotum* and *Wikstroemia pseudoretusa* were found only by using microhistological analysis. In addition, the shells of snails (Pulmonata) and arthropods, which were not targeted by the DNA barcoding, were observed by microhistological analysis.

**Table 2:** List of the lowest taxonomic levels in the diet of the red-headed wood pigeon and its presence in DNA barcoding and microhistological analysis

Food items	Native/Introduced	*N* reads	*F*_S_ (%)	*F*_O_ (%) DNA barcoding	*F*_O_ (%) microhistology
Plants					
*Morus australis*	Introduced	11,810	36.58	95.83	0.00
Gr. Lauraceae1	Native	11,280	34.94	70.83	59.57
Gr. Ficus1	Native/Introduced	3902	12.08	93.75	4.00
*Fagara boninsimae*	Native	1328	4.11	33.33	31.91
Gr. Poaceae2	Introduced	922	2.86	66.67	0.00
*Ligustrum micranthum*	Native	514	1.59	6.25	0.00
*Leucaena glauca*	Introduced	335	1.04	12.50	0.00
*Sambucus javanica*	Introduced	297	0.92	20.83	0.00
*Carex hattoriana*	Native	231	0.72	2.08	0.00
*Ardisia sieboldii*	Native	226	0.70	18.75	0.00
*Trema orientalis*	Native	212	0.66	4.17	0.00
Gr. Planchonella1	Native	158	0.49	12.50	12.77
Poaceae	Introduced	115	0.36	31.25	0.00
Moraceae	Native/Introduced	114	0.35	14.58	0.00
Gr. Palmae2	Native/Introduced	90	0.28	31.25	0.00
*Elaeocarpus photiniifolius*	Native	87	0.27	10.42	2.00
*Lantana camara*	Introduced	77	0.24	18.75	6.38
*Buxus liukiuensis*	Introduced	72	0.22	6.25	0.00
*Rhaphiolepis wrightiana*	Native	67	0.21	8.33	0.00
*Solanum nigrum*	Introduced	63	0.20	2.08	0.00
*Juniperus taxifolia*	Native	62	0.19	4.17	0.00
*Calaphyllum inophyllum*	Native	51	0.16	10.42	0.00
*Sonchus oleraceus*	Introduced	43	0.13	4.17	0.00
*Schima mertensiana*	Native	41	0.13	10.42	0.00
*Eurya boninensis*	Native	23	0.07	6.25	0.00
*Celtis boninensis*	Native	21	0.07	10.42	0.00
*Derris elliptica*	Introduced	20	0.06	6.25	0.00
*Hedyotis grayi*	Native	18	0.06	2.08	0.00
Rutaceae	Native	17	0.05	4.17	0.00
*Paederia scandens*	Introduced	13	0.04	4.17	0.00
*Livistona chinensis*	Native	10	0.03	4.17	2.00
Gr. Myrtaceae1	Native/Introduced	10	0.03	2.08	0.00
*Scaevola sericea*	Native	9	0.03	4.17	0.00
*Carica papaya*	Native	9	0.03	2.08	0.00
*Pinus luchuensis*	Introduced	7	0.02	2.08	0.00
*Terminalia catappa*	Native	7	0.02	2.08	0.00
*Osmanthus insularis*	Native	6	0.02	4.17	0.00
*Pennisetum purpureum*	Introduced	5	0.02	2.08	0.00
*Drypetes integerrima*	Native	4	0.01	2.08	0.00
*Elaeagnus rotundata*	Native	4	0.01	2.08	0.00
*Melastoma tetramerum*	Native	4	0.01	2.08	0.00
*Paspalum conjugatum*	Introduced	4	0.01	2.08	0.00
*Distylium lepidotum*	Native	0	0.00	0.00	2.00
*Wikstroemia pseudoretusa*	Native	0	0.00	0.00	2.00
Animals					
Plumonata	Native/Introduced	–		–	0.15
Arthropoda	Native/Introduced	–		–	0.02

### Monthly change of diet composition and difference between the islands, as estimated by DNA barcoding

**Figure 4:**
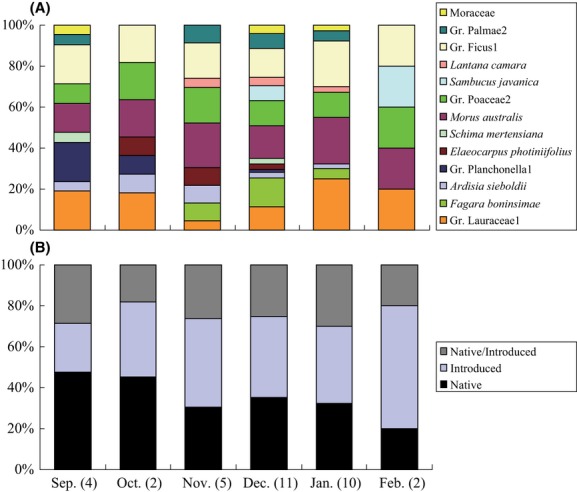
Monthly relative frequencies of presence data for major food plants during the breeding season on Chichijima, based on DNA barcoding (A). The numbers in parentheses show the sample sizes for each month. Each plant sequence group is designated as a native species, as an introduced species, or as containing both native and introduced species (B).

**Figure 5:**
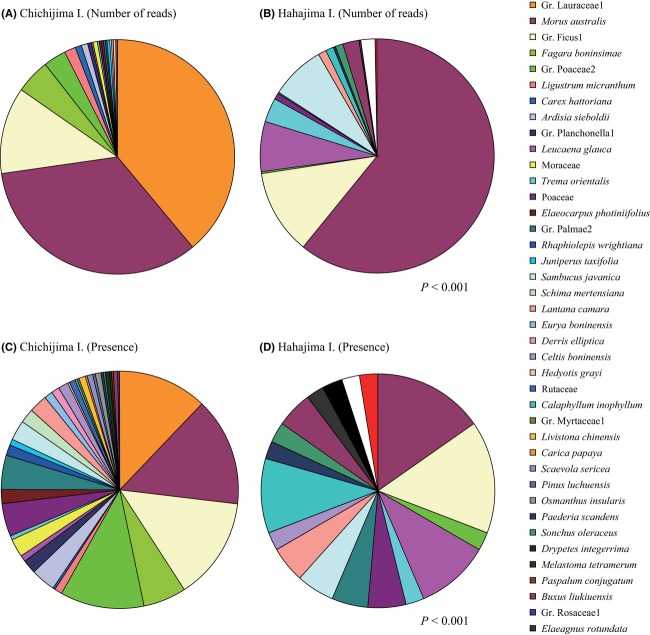
Comparison of diet composition between Chichijima Island and Hahajima Island based on the frequency of reads (A and B) and the relative frequency of presence data (C and D) for each food item.

**Figure 6:**
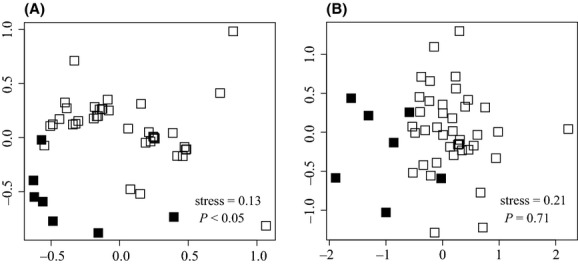
The results of the similarity analysis with NMDS and ANOSIM, based on presence/absence (A) and number of reads (B). White squares designate samples from Chichijima, and black squares designate samples from Hahajima.

Figure 4 shows the monthly change in major food plants (detected in more than 10% of the samples) during the breeding season in Chichijima. The native species *Fagara boninsimae* and *Planchonella* were frequently observed only during specific months (December and September, respectively), whereas native Lauraceae, introduced *Morus australis,* and Gr. Poaceae2 were consistently observed at high frequencies throughout the breeding season. There were significant differences between the dietary compositions of pigeons on Chichijima and those on Hahajima in both of the estimations (frequency of reads and relative frequency of presence data; Fig. 5). The results of the similarity analysis by NMDS and ANOSIM demonstrated the significant differences between the diet compositions on Chichijima and Hahajima based on the presence/absence data for each plant taxa in each sample (*R* = 0.2236, *P* < 0.05, Fig. 6), and there was no significant difference according to the analysis based on the number of reads.

## Discussion

### Applicability of DNA barcoding for diet analysis of the red-headed wood pigeon

The DNA barcoding method via *trn*L P6 loops produced a much higher resolution than microhistological analysis, as reported by some previous studies (Soininen et al. 2009; Valentini et al. 2009a,b; Raye′ et al. 2011). The comprehensiveness of the P6 loop database used in the present study appeared to be sufficient given the high rate of food plant identification from the pigeon feces. This finding may be explained by the high endemism and low species richness of the oceanic island flora, which may improve the comprehensiveness of the local sequence database and can be a major limitation for food identification using the DNA barcoding approach (Valentini et al. 2009a). The results of the present study indicate the clear advantage of DNA barcoding over microhistological analysis, the latter of which has a bias favoring easily identified food items (Soininen et al. 2009). Lauraceae, *Fagara boninsimae,* and *Planchonella*, which were frequently observed using both methods, have hard and large seeds. Thus, these plants may be easy to identify using microhistological analysis because larger fragments may remain in feces, even after being crushed in a pigeon's stomach. In contrast, *Morus australis*, *Ficus,* and Poaceae, which were frequently observed only by DNA barcoding, have soft and small seeds and thus may be difficult to identify using microhistological analysis; furthermore, most of these plants are introduced species (except for three native species belonging to Gr. Ficus1). These results indicate the underestimation of the pigeons' use of introduced plants in previous microhistological analyses (Shibazaki and Hoshi 2006).

However, some limitations of the DNA barcoding approach have also been presented. The first problem with this technique is its low discrimination rate within the P6 loop database for specific families (Lauraceae and Moraceae at 14% and 20%, respectively, Table 1). In the case of Lauraceae, *Neolitsea aurata* was detected using microhistological analysis, although this species cannot be distinguished from the other Lauraceae species using P6 loop sequences. The performance of DNA barcoding will be improved by hierarchical barcoding (Moszczynska et al. 2009) using family-specific barcoding markers. The second problem is that some food items identified by microhistological analysis were not observed using DNA barcoding (Table 2). In the case of the plants *Distylium lepidotum* and *Wikstroemia pseudoretusa*, there were no sequence mismatches in the primer-binding sites, which may have caused the strong amplification efficiency bias (Deagle et al. 2007). Thus, the inability to detect these plants may have been caused by a failure in the DNA extraction rather than a failure in the PCR amplification. These bias errors can occur when a DNA extraction sample is obtained from a single scat, particularly for dietary analysis of large mammals (Deagle et al. 2005). Although this bias may be small in the fecal analysis of small bird scat, the appropriate extraction strategies (e.g., subsampling and blending, Deagle et al. 2009; Kowalczyk et al. 2011) should be considered. The observation of snails and arthropods in the microhistological analysis indicates that the pigeons ate small animals; thus, not only plants were targeted in the DNA barcoding method. Diet analysis using animal-targeted markers (e.g., COI, Meusnier et al. 2008) may provide more detailed information regarding pigeon ecology. This result also suggests the importance of microhistological analysis for understanding the overall diet, which is essential for choosing an appropriate barcode (Pompanon et al. 2012).

### Monthly changes in diet composition on Chichijima

These results indicate the pigeons' frequent consumption of not only native species but also introduced species. The native *Fagara boninsimae* and *Planchonella* species seem to be consumed at maximum fruiting times (Toyoda 2003). The consistent presence of Lauraceae may be explained by the long fruiting period of *Neolitsea aurata* (Hayashi 1985) and to the sequence encoded by several species that exhibit different fruiting phenologies (e.g., *Neolitsea aurata*, *Machilus kobu,* and *Machilus boninensis*, Toyoda 2003). The results may also indicate the pigeons' preference for Lauraceae species. This finding is supported by direct observations (Kanto Regional Environmental Office 2011) and frequent feeding by other (sub)species, including the Japanese wood pigeon *Columba janthina janthina* (Wild Bird Society of Japan 1998) and the Madeira laurel pigeon *Columba trocaz* (Oliveira et al. 2002). The fruiting periods of the introduced plants *Morus australis* and Poaceae are long and irregular (Toyoda 2003; Ando personal observation), and thus, these plants could be used throughout the breeding season. Although the P6 loop database could not identify the introduced plant *Ficus microcarpa* or other native *Ficus* species, the frequently observed sequence for Gr. Ficus1 may belong to the introduced *Ficus microcarpa*, considering the observation records of pigeon feeding and the great fruiting abundance of this plant in the Ogasawara Islands (Ecological Society of Japan 2002; Shibazaki and Hoshi 2006). Owing to a lack of sufficient native food resources, the red-headed wood pigeon may depend on these introduced species.

In the Ogasawara Islands, the eradication of introduced plants has been conducted rapidly over the last decade to conserve the endemic ecosystems (e.g., *Bischofia javanica*, Tanaka et al. 2010). *Ficus microcarpa* has recently been growing in numbers after the introduction of the pollinator *Eupristina verticillata* (Yokoyama 1996; Ecological Society of Japan 2002) and may also be eradicated in the future as a result of its negative impacts on native ecosystems (Ecological Society of Japan 2002; Watanabe et al. 2009). To conserve the habitat of the red-headed wood pigeon, the direct and indirect effects of introduced species eradication (Bergstrom et al. 2009; Simberloff et al. 2013) should be considered. The rapid eradication of introduced species may reduce the food resources for the pigeon and may have a negative impact on the pigeon population. However, native flora can provide various fruits throughout the year, and these plants may recover after eradicating the introduced species. To facilitate the long-term survival of the red-headed wood pigeon, using the various native fruits rather than specific dominant introduced fruits may be more effective in terms of food quality and quantity. However, the present study could not reveal whether the pigeons selectively use the detected food plants or just consumed what is available. An estimation of food resource availability and quality (e.g., nutrients) may provide a better understanding of the impact of introduced species eradication on the pigeon population.

### Composition differences in pigeon diets between the islands

Comparing the pigeon food compositions based on the number of reads and presence data indicated significant differences between Chichijima and Hahajima (Figs. 5, 6A). Although *Morus australis* and *Ficus* dominated both of the islands, the results may indicate different patterns of food selection by the pigeons on each of the islands. The native Lauraceae species was observed only in Chichijima at a high frequency. This finding may be explained by its significant fruiting abundance in the forest of Chichijima (Toyoda 2003), indicating its importance as a major native food resources on the island. In Hahajima, the read percentage of introduced *Morus australis* was much larger than that of Chichijima. These data may indicate the greater usage of this introduced species by the pigeons in Hahajima than those of Chichijima. *Leucaena leucocephala*, which exhibited the third highest frequency in terms of both read numbers and presence in Hahajima, is also an expanding introduced species in the Ogasawara Islands (Hata et al. 2010). This plant lives on the forest edge (Toyoda 2003), which is not the preferred forest habitat of the pigeon. Pigeons on Hahajima may eat *Leucaena leucocephala* to make up for a lack of food resources in the forest area. It is also interesting that the food composition varied among Hahajima samples (Fig. 6), despite the small sample size and restricted sampling site (five samples were collected in February around the same roost). The red-headed wood pigeons in Hahajima are more generalist than those of Chichijima because of a lack of food resources. Estimations of food resource availability and temporal diet variation on each island may reveal differences in pigeon feeding strategies between these locations.

We also compared data from the number of reads and the relative frequency of presence for each plant taxa. The reliability of quantitative diet analysis using the sequence count data has been discussed in several previous studies (e.g., Deagle et al. 2009, 2010; Soininen et al. 2009; Valentini et al. 2009a; Raye′ et al. 2011; Pompanon et al. 2012; Shehzad et al. 2012). The PCR efficiency and resulting number of sequences from each food item can be affected by several biological (e.g., number of DNA copies in a unit mass of tissues, digestion process) and technical factors (e.g., different sequence lengths among species, mismatches in primer-binding sites; Pompanon et al. 2012). Indeed, one of the shortest plant sequences dominates the dietary analysis of the alpine chamois *Rupicapra rupicapra* in Raye′ et al. (2011). Thus, Pompanon et al. (2012) recommended using the read count data for measuring spatial or temporal variations in the diet but not for absolute diet quantification. In the case of the present study, comparing the proportion of each plant read number between the two islands can be reliable (e.g., sequence proportion of *Morus australis*). The sequence lengths of the Gr. Lauraceae1 (87 bp, only found in Chichijima), *Morus australis* (89 bp), and Gr. Ficus1 (89 bp), which exhibited high-frequency read numbers, were not much shorter than those of the other detected food plants (ranging 72-121 bp, average 89.9 bp), and their frequencies of presence were also high. Thus, pigeons may have actually eaten these plants in large amounts, as reported by Deagle et al. (2010) regarding the accuracy of quantitative diet data from the number of reads. However, the pigeon consumption of some plants with low PCR efficiency may be underestimated in the analysis based on the number of reads. For example, the low sequence proportion of Gr. Poaceae2 in Chichijima (4%) may be explained by its low PCR efficiency, despite its high frequency (75%) (Fig. 5A and C). No significant difference between Chichijima and Hahajima in the NMDS analysis was based on the number of reads, which may reflect the dominance of specific plant sequences (e.g., *Morus australis* and Gr. Ficus1) throughout the samples and the underestimation of some plants with low PCR efficiency. Another possibility is that the small number of reads for some plants may indicate secondary predation (Sheppard et al. 2005), such as plants eaten by snails or other invertebrate herbivores eaten by pigeons, or that the number of reads reflects the biomass to some extent (Yoccoz et al. 2012). This is an important issue to be considered in estimating pigeon food preferences. Given that appropriate methods to correct various biases in quantitative analysis have never been presented, it is advisable to use not only the number of reads but also the frequency of presence to estimate dietary composition and diversity, as in Soininen et al. (2009), Raye′ et al. (2011), and Shehzad et al. (2012).

## Conclusions

In this study, a diet analysis using DNA barcoding provided a high-resolution identification of food plants and clearly overcame the bias of traditional microhistological analysis. The results of the DNA barcoding indicated frequent consumption of introduced species, rather than only native species, by the pigeons. The rapid eradication of some introduced species without restoration of the native seed plants may reduce available food resources for this pigeon. Thus, a strategy that balances the eradication of introduced plants and the restoration of native food resources is important. Differences between the composition of pigeon diets on Chichijima and those on Hahajima should also be considered during the restoration of each island. Although some existing technical problems must still be solved (e.g., the discrimination rate of the P6 loop database, sampling strategy), the NGS DNA barcoding approach will provide a better understanding of the food web, including the interactions between native and introduced species and appropriate nature restoration planning for oceanic island ecosystems.

## Data Accessibility

DNA sequences of chloroplast trnL P6 loop database can be found in the DDBJ accessions AB817341-AB817701.

A FASTA file of the fecal sequence will be archived in the Dryad repository.

## Supporting Information

**Appendix S1.** Species groups representing the lowest taxonomic levels in the P6-loop database and their associated families.
