# Supplementary material for: Diet analysis by next-generation sequencing indicates the frequent consumption of introduced plants by the critically endangered red-headed wood pigeon (Columba janthina nitens) in oceanic island habitats
Source: Ecol Evol. 2013 Sep 19;3(12):4057–69. doi: 10.1002/ece3.773 (PMC3853553; doi:10.1002/ece3.773)
Supplement: Supplementary file 1 [file ece30003-4057-SD1.docx]

**Supporting information**

**Appendix 1** Species groups representing the lowest taxonomic levels in the P6-loop database and their associated families.

|  |  |  |
| --- | --- | --- |
| Group | Species | Family |
| Gr. Agavaceae1 | *Dracaena spp.* | Agavaceae |
|  | *Sansevieria nilotica* | Agavaceae |
|  | *Vernonia cinerea var. parviflora* | Asteraceae |
|  | *Youngia japonica* | Asteraceae |
|  | *Youngia japonica* | Asteraceae |
| Gr. Callicarpa1 | *Callicarpa glabra* | Verbenaceae |
|  | *Callicarpa nishimurae* | Verbenaceae |
|  | *Callicarpa subpubescens* | Verbenaceae |
| Gr. Citrus1 | *Citrus limon* | Rutaceae |
|  | *Citrus maxima* | Rutaceae |
|  | *Citrus sinensis* | Rutaceae |
| Gr. Cyperaceae1 | *Cyperus kyllingia* | Cyperaceae |
|  | *Schoenus brevifolius* | Cyperaceae |
| Gr. Cyperaceae2 | *Carex hattoriana* | Cyperaceae |
|  | *Carex oahuensis* | Cyperaceae |
|  | *Carex toyoshimae* | Cyperaceae |
| Gr. Cyperaceae3 | *Cyperus cyperoides* | Cyperaceae |
|  | *Cyperus microiria* | Cyperaceae |
| Gr. Diospyros1 | *Diospyros ferrea* | Ebenaceae |
|  | *Diospyros kaki* | Ebenaceae |
| Gr. Ficus1 | *Ficus benghalensis* | Moraceae |
|  | *Ficus boninsimae* | Moraceae |
|  | *Ficus elastica* | Moraceae |
|  | *Ficus iidaiana* | Moraceae |
|  | *Ficus microcarpa* | Moraceae |
|  | *Ficus nishimurae* | Moraceae |
|  | *Ficus religiosa* | Moraceae |
|  | *Ficus superba* | Moraceae |
| Gr. Hedyotis1 | *Hedyotis grayi* | Rubiaceae |
|  | *Hedyotis mexicana* | Rubiaceae |
| Gr. Hibiscus1 | *Hibiscus glanber* | Malvaceae |
|  | *Hibiscus tiliaceus* | Malvaceae |
| Gr. Ilex1 | *Ilex beecheyi* | Aquifoliaceae |
|  | *Ilex matanoana* | Aquifoliaceae |
| Gr. Lauraceae1 | *Cinnamomum pseudo-pedunculatum* | Lauraceae |
|  | *Machilus kobu* | Lauraceae |
|  | *Machilus pseudokobu* | Lauraceae |
|  | *Neolitsea aurata* | Lauraceae |
|  | *Neolitsea boninensis* | Lauraceae |
| Gr. Melastoma1 | *Melastoma tetramerum* | Melastomataceae |
|  | *Melastoma tetramerum var. pentapetalum* | Melastomataceae |
| Gr. Miscanthus1 | *Miscanthus boninensis* | Poaceae |
|  | *Miscanthus condensatus* | Poaceae |
| Gr. Morinda1 | *Morinda boninensis* | Rubiaceae |
|  | *Morinda umbellata var. hahazimensis* | Rubiaceae |
| Gr. Myrsine1 | *Myrsine maximowiczii* | Myrsinaceae |
|  | *Myrsine okabeana* | Myrsinaceae |
| Gr. Myrtaceae1 | *Psidium cattleianum* | Myrtaceae |
|  | *Syzygium* | Myrtaceae |
|  | *Syzygium buxifolium* | Myrtaceae |
|  | *Syzygium cleyeraefolium* | Myrtaceae |
| Gr. Palmae1 | *Arenga engleri* | Palmae |
|  | *Caryota urens* | Palmae |
|  | *Chrysalidocarpus lutescens* | Palmae |
|  | *Mascarena verschaffeltii* | Palmae |
|  | *Ptychosperma elegans* | Palmae |
|  | *Veitchia merrillii* | Palmae |
| Gr. Palmae2 | *Clinostigma savoryana* | Palmae |
|  | *Neodypsis decaryi* | Palmae |
| Gr. Pittosporum1 | *Pittosporum chichijimense* | Pittosporaceae |
|  | *Pittosporum parvifolium* | Pittosporaceae |
|  | *Pittosporum beecheyi* | Pittosporaceae |
|  | *Pittosporum boninense* | Pittosporaceae |
| Gr. Planchonella1 | *Planchonella boninensis* | Sapotaceae |
|  | *Planchonella obovata* | Sapotaceae |
|  | *Planchonella obovata var. dubia* | Sapotaceae |
| Gr. Poaceae1 | *Isachne* spp. | Poaceae |
|  | *Oplismenus compositus* | Poaceae |
|  | *Paspalum urvillei* | Poaceae |
| Gr. Poaceae2 | *Arundo donax* | Poaceae |
|  | *Echinochloa crusgalli* | Poaceae |
|  | *Paspalum notatum* | Poaceae |
|  | *Paspalum scrobiculatum* | Poaceae |
| Gr. Rosaceae1 | *Osteomeles boninensis* | Rosaceae |
|  | *Osteomeles lanata* | Rosaceae |
|  | *Photinia wrightiana* | Rosaceae |
| Gr. Rutaceae1 | *Boninia crassifolia* | Rutaceae |
|  | *Boninia glabra* | Rutaceae |
|  | *Boninia grisea* | Rutaceae |
|  | *Evodia nishimurae* | Rutaceae |
| Gr. Stachyuraceae1 | *Stachyrus macrocarpus* | Stachyuraceae |
|  | *Stachyrus macrocarpus* var. *prunifolius* | Stachyuraceae |
| Gr. Symplocos1 | *Symplocos kawakamii* | Symplocaceae |
|  | *Symplocos pergracilis* | Symplocaceae |
|  |  |  |
